# Determine the impact of a structured pharmacist-led medication review - a controlled intervention study to optimise medication safety for residents in long-term care facilities

**DOI:** 10.1186/s12877-022-03025-3

**Published:** 2022-04-09

**Authors:** M. Lexow, K. Wernecke, R. Sultzer, T. Bertsche, S. Schiek

**Affiliations:** 1 Department of Clinical Pharmacy, Institute of Pharmacy, Faculty of Medicine, Leipzig University, Bruederstr. 32, 04103 Leipzig, Germany; 2 Drug Safety Center, University Hospital of Leipzig and Leipzig University, Leipzig, Germany; 3 Sana Geriatric Hospital Zwenkau, Pestalozzistr. 9, 04442 Zwenkau, Germany

**Keywords:** Medication review, Drug related problem, Older patient, Long-term care facility, Medication safety, Pharmaceutical intervention

## Abstract

**Background:**

Medication reviews contribute to protecting long-term care (LTC) residents from drug related problems (DRPs). However, few controlled studies have examined the impact on patient-relevant outcomes so far.

**Objective:**

We examined the impact of a one-time, pharmacist-led medication review on medication changes (primary endpoint) including discontinued medication, the number of chronic medications, hospital admissions, falls, and deaths (secondary endpoints).

**Methods:**

A prospective, controlled intervention study was performed in three LTC facilities. In the intervention group (IG), after performing a medication review, a pharmacist gave recommendations for resolving DRPs to physicians, nurses and community pharmacists. The control group (CG) received usual care without a medication review. (i) We assessed the number of medication changes and the secondary endpoints in both groups before (t0) and after (t1, t2) the intervention. (ii) Additionally, the medication review was evaluated in the IG with regard to identified DRPs, the healthcare professional’s feedback on the forwarded pharmacist recommendations and whether DRPs were finally resolved.

**Results:**

107 (IG) and 104 (CG) residents were enrolled. (i) More medication changes were identified in the IG than in the CG at t1 (*p* = 0.001). However, no significant difference was identified at t2 (*p* = 0.680). Mainly, medication was discontinued in those medication changes. Chronic medications increased in the CG (*p* = 0.005) at t2 while hospital admissions, falls, and deaths showed no differences. (ii) Overall, 1252 DRPs (median: 10; minimum-maximum: 2–39) were identified. Recommendations for 82% of relevant DRPs were forwarded to healthcare professionals, of which 61% were accepted or clarified. 22% were not accepted, 12% required further review and 6% remained without feedback. 51% of forwarded DRPs were finally resolved.

**Conclusions:**

We found more medication changes in the IG compared to controls. Mostly, medication was discontinued. This suggests that our intervention was successful in discontinuing unnecessary medication. Other clinical outcomes such as falls, hospitalisations, and deaths were not improved due to the one-time intervention. The medication review further identified a high prevalence of DRPs in the IG, half of which were finally resolved.

**Trial registration:**

German Clinical Trials Register, DRKS00026120 (www.drks.de, retrospectively registered 07/09/2021).

## Introduction

Residents in long-term care (LTC) facilities often have multiple chronic disease states [1] that increase their likelihood for polypharmacy and complex therapy regimes [2, 3]. This leads to an increased risk for drug-related problems (DRPs) in the LTC setting for 75% of residents [4, 5]. A DRP is defined as an event or circumstance involving drug therapy that actually or potentially interferes with desired health outcomes [6]. Negative outcomes such as adverse drug reactions, hospital admissions, mortality and related high costs can affect both the individual patient and the healthcare system [7–10].

An effective strategy to enhance the quality of drug therapy and to resolve DRPs is to involve pharmacists in a multi-professional collaboration with physicians and nurses by providing medication reviews [11, 12]. The Pharmaceutical Care Network Europe (PCNE) defined the term ‘medication review’ as follows: “Medication review is a structured evaluation of a patient’s medicines with the aim of optimising medicines use and improving health outcomes. This entails detecting drug-related problems and recommending interventions” [13]. Different types of medication reviews are performed in routine, such as comprehensive medication reviews (annually recommended) or targeted medication reviews (quarterly recommended) [14]. Randomised controlled studies that have carried out a one-time medication review in the LTC setting have also already proven the benefit of those pharmaceutical services by systematically assessing residents’ medication records in terms of identified DRPs [15, 16]. Thereby, pharmacists provide recommendations for medication changes, such as discontinuation of inappropriate medication, changes of dose, or modifications of drug regimens. This results in enhanced medication appropriateness as well as positive economic outcomes [17]. However, little is known about the influence of medication reviews on patient-relevant outcomes (e.g., hospital admissions or mortality) [18]. In addition, the number of medication changes can be a valuable parameter for measuring successful interventions [19], especially when they are associated with deprescribing of inappropriate medications [20]. Therefore, an increased medication change rate indicates a greater attentiveness to patient’s medication-related needs [21].

The routine implementation of pharmacy services in LTC facilities varies among countries worldwide. In Australia and the United States, pharmacists’ medication reviews in LTC facilities are already implemented and government-funded [22, 23]. In many other countries, including Germany, these services are neither routinely established nor remunerated [24, 25]. In German LTC facilities, general physicians (GPs) and specialists usually are responsible for the diagnosis and prescription of medication; the nursing staff organise the medication process and the pharmacies supply the medication. So far, no uniform regulations exist for comprehensive medication reviews or multi-professional collaboration of the healthcare professionals involved in this setting. To achieve the goal of evaluating the acceptability of medication change proposals, it is necessary to involve all relevant professional groups in interventions. Data that support implementation measures in various settings are valuable for a broader application of pharmaceutical services in the LTC setting.

Therefore, this study aimed to investigate the impact of a one-time, pharmacist-led medication review (intervention group, IG) that resolved DRPs on medication changes and further patient-relevant outcomes compared to a control group (CG) who received usual care.

## Methods

### Setting

The multicentre study was conducted in three LTC facilities with different ownership (welfare, municipal and private associations) in the area of Leipzig, Germany. A total of 11 units across the three facilities participated in the study. The physicians (GPs and specialists) were not employed by the LTC facilities and visited the facilities periodically with variable frequencies; within these visits, they had access to the documentation of residents’ medical records*.* Community pharmacists in this setting were focused on the logistical delivery of medications.

### Study design

A prospective, controlled intervention study was performed. Data were collected consecutively, first in the CG and second in the IG. A consecutive instead of a simultaneous design was chosen because the same nurses, physicians and pharmacies were involved in both study groups and would have been influenced by an intervention in the CG.

In addition, this population has a high mortality rate. Therefore, randomisation of residents at baseline was not possible, as we delayed the beginning of the IG until after the CG. Nevertheless, we allowed random assignment of participants into both groups by selecting the residents’ rooms before data collection. Therefore, the rooms of each participating ward were randomly assigned by one of the study pharmacists (ML) to CG or IG using a computer-generated simple random sequence (www.random.org).

### Recruitment

During each study period, residents who lived in the respective room and fulfilled the inclusion criteria were invited (directly or through their legal representatives) by one of the researchers (ML) to participate in the study. Additionally, all attending physicians and involved community pharmacies were prior informed (by ML) about the study, either in person or in writing.

### Inclusion and exclusion criteria

The following inclusion criteria were required at time of recruitment: age ≥ 65, long-term/chronic medicines (without counting pro re nata (PRN) medications) ≥ 3, multimorbidity with ≥3 comorbidities and written informed consent. Different from the common definition of polypharmacy, which is often associated with the use of five or more medications, we opted for the inclusion criterion of at least three medications. This decision was supported by a previous study by our research group in which the prevention of DRPs was investigated in geriatric patients in a comparable setting [26]. Clinically relevant DRPs could occur with at least three concomitantly administered drugs. For example, before forwarding a drug-drug interaction recommendation to a physician, clinical relevance was weighed in terms of the therapeutic benefit and risks for potential harm based on a commonly used database. In this process, only recommendations that were found to be clinically relevant were forwarded to the physician after considering patient-related data from clinical values and laboratory data as well as drug-related data from the database. Furthermore, a large number of residents should be included who could benefit from this previously unestablished service. Residents were excluded if life expectancy was assessed less than 6 months according to present health information or if participation was declined.

### Data collection

In both study groups, data collection comprised 3 time points for data assessment: baseline (t0), a first (t1) and a second (t2) follow-up. While the CG received usual care without a medication review, a one-time, pharmacist-led medication review per resident was carried out only in the IG between t0 and t1. No intervention was performed between t1 and t2. The aim was to keep the time intervals between the measurement points as equal as possible under routine conditions. Due to the higher organisational effort in the IG, the time interval between t0 and t1 ranged in both groups (6 weeks to 3 months). The time interval between t1 and t2 was the same in both groups (3 months). The study was conducted over a time period of 2 years and 2 months (time period in the CG: 10 months; time period in the IG: 16 months).

In both groups, two pharmacists of the research group (ML, KW) performed a standardised data collection of residents’ health and medication information including residents’ medical records; whereas, the medication review in the IG was performed by one of the pharmacists (ML). To ensure a standardised approach, data collection was conducted by both pharmacists together during a training phase at the beginning of the study. All data were collected in the LTC facilities and covered information about demographic characteristics, diagnosis, laboratory data, relevant vital parameters, hospital admissions, fall events and deaths. The current medication therapy (chronic and PRN medication) was taken from the medication charts of LTC facilities, which were either written or digital based. In addition to t0, at t1 and t2 the medication changes were further collected with details of actual changes in subcategories (e.g., stopped drugs, new drugs, dose changes, documentation changes in the medication charts) and compared to the previous medication chart.

### Outcomes

#### Outcome comparison

For the comparison of CG and IG, the number of medication changes in residents’ records was examined as the primary outcome. Secondary outcomes were the number of medication changes in subcategories, chronic medications, hospital admissions, falls, and deaths.

#### Medication review procedure

An evaluation of the medication review procedure was performed in the IG regarding: (a) DRP identification, (b) pharmacist recommendations, (c) the healthcare professional’s feedback on pharmacist recommendations and (d) resolved DRPs.

### Control group

Participants in the CG received usual care without an additional structured medication review initiated by a pharmacist as conducted in the IG.

### Intervention procedure: structured medication review

The medication review was performed by one of the pharmacists of the research group (ML, PhD student) who had completed advanced training in geriatric pharmacy and was experienced (2.5 years) in supplying LTC residents. The medication review procedure comprised the identification of DRPs including evaluation of the relevance, forwarding clinically relevant pharmacist recommendations to healthcare professionals and determining healthcare professionals’ feedback. Finally, pharmacist recommendations were determined in terms of actually implemented recommendations in order to assess resolved DRPs.

For the medication review, all available patient and medication-related information was accessed by the research pharmacist through review of residents’ medical records, including the medication charts in the LTC facilities, and considering the following items: prescribed medications, diagnosis, relevant laboratory data (e.g., renal status) and relevant physical parameters (e.g., weight, blood pressure). To enable a standardised procedure, a DRP checklist was used. Items related to the ‘prescribing problems’ were based on standard classification systems [6, 27, 28] that were adapted for this setting by including an additional category of potentially inappropriate medication (PIM) for older persons [29, 30]. These categories were completed in categories that covered ‘medication use problems’ and ‘documentation problems’. Table 1 shows all DRP categories and criteria for evaluation.

**Table 1:** Explanation of DRP categories and evaluation criteria

DRP categories	Evaluation criteria
***Prescribing problems***	
Contraindication	- Medication is generally not recommended according to the SmPCs by assessing documented diagnosis.
Therapeutic duplication	- Consideration of duplicate active drug or duplicate drug class.
Dosing problems	- Dose is not recommended (too low, too high) or not adjusted for age, weight or renal failure according to the SmPCs.
Drug-drug interaction	- Interaction described by the database used and considered as clinically relevant by using all available clinical parameters (e.g., laboratory data).
Inappropriate administration time	- Administration time is not recommended according to the SmPCs.
Inappropriate drug form	- Drug form is not appropriate for residents’ intake practices (e.g., chewing medication).
Inappropriate treatment duration	- Treatment duration is not recommended (too long, too short) according to the SmPCs.
Inappropriate medication for indication or concomitant condition	- A medication is prescribed that is not the recommended medication choice according to clinical therapy guidelines for the documented diagnosis or concomitant condition.
Insufficient drug treatment	- Additional medication is required according to clinical therapy guidelines by assessing the documented diagnosis.
Potentially inappropriate medication	- Medications with increased risk for adverse effects according to the Beers list [29]/ Priscus list (German PIM list) [30].
Unclear indication for drug	- Medication without a clear indication according to the SmPCs is used by assessing documented diagnosis.
***Medication use problems***	
Incorrect storage	- Missing medication package according to the medication chart; Lack of recommended resident name reference on medication package; Usage of medication after expiration date.
Incorrect dispensing	- Comparison of residents medication charts with the current dispensed medication (in cups or dose administration aids prepared for administration in advance) in terms of wrong medication, wrong dose, omission, additional not prescribed medication, wrong time, not recommended division of tablets or not recommended removal from primary packaging according to the SmPCs.
Incorrect preparation	- Not recommended crushing of tablets or opening of capsules according to the SmPCs; Crushing of several tablets at the same time; Mixing of crushed and liquid medications.
Incorrect administration	- Comparison of residents medication charts with current medication administered to the residents in terms of wrong resident, wrong medication, omission, additional not prescribed medication, wrong time, no attention to individual intake problems (e.g., chewing the medication), which would require a different preparation.
***Documentation problems***	
Incomplete or unclear documentation in the medication records	- No clear identification of the medication; No clear identification of dose or drug form when several are available; Wrong or missing information for required drug preparation or administration (e.g., crushing of tablets); Missing information for PRN medication (e.g., no minimal or maximal dose); Discrepant information (e.g., more than one medication chart available with discrepant information for medications).

*Abbreviations*: *PIM* Potentially inappropriate medication*, PRN* pro re nata, *SmPC* Summary of Product Characteristics

First, to identify DRPs, residents’ medication records were reviewed at the prescribing level according to the predefined DRP checklist. Drug information was obtained from the summary of product characteristics (SmPCs), a drug-drug interaction database used in public pharmacies (ABDA Database, ABDATA, Eschborn, Germany) and clinical therapy guidelines. Additionally, we considered DRPs at the medication use level by including further information about storage, documentation, dispensing as well as preparation and administration of medication. Therefore, nursing staff was observed in preparation (e.g., modification of medication, including crushing tablets or mixing medications) and administration of medication (e.g., verification of the right medication for the right resident and at the right time) during a morning drug round, once per resident. Drug-taking habits and health-related information, such as swallowing difficulties, were also collected from residents’ medication records and by a structured questioning of the nursing staff. Furthermore, therapy and documentation in the residents’ medication records were compared with stored and dispensed drugs. Documentation problems in the medication records were recorded in terms of correctness and completeness, such as missing information for PRN medication. All identified DRPs were evaluated in regard of the clinical relevance. Not relevant DRPs were excluded considering all available sources and clinical parameters. For example, the interaction of two antihypertensive drugs, which only needs to be considered at the beginning, could be neglected in long-term use where these additive effects may even be desired. Therefore, these DRPs were not classified as clinically relevant and not further forwarded to the physicians. Since PRNs could also potentially have been administered and thus pose a risk for the occurrence of DRPs, such as drug-drug interactions, we considered all prescribed PRN and chronic medications for the medication review.

Secondly, recommendations deemed relevant to resolving the identified DRPs were compiled. These included suggestions for changes such as discontinuing a medication without a clear indication or recommendations in the absence of documentation in medication records. To ensure consistency, accuracy, and relevance, the DRPs and recommendations were checked by a second experienced research pharmacist (SC). In cases of uncertainty, the DRPs were discussed until agreement was reached.

Third, the involved physicians (GPs and specialists), nursing staff and the community pharmacies were contacted, and the relevant pharmacist recommendations were forwarded to them and reconsidered in a multi-professional exchange. The healthcare professionals’ feedback was collected in regard of the acceptance of pharmacist recommendations. For the physician contact, a brief explanation of the scientific background and the sources used was added in written form. The nursing staff was usually contacted first and further relevant information to resolve the DRPs was collected, such as whether PRN medications were actually administered. Pharmacist recommendations concerning the nursing staff (e.g., storage) were discussed directly. Then, DRPs were forwarded to the physicians and, if necessary, also to the supplying pharmacies (e.g., supply issues). First choice of contact was a personal face-to-face meeting. Personal contact with nursing staff took place in the LTC facilities, with the physicians in their medical practices and with the community pharmacies in their pharmacies. If this was not possible, they were contacted by phone or in writing. Although contact with different healthcare professionals was possible for a DRP, the healthcare professional (physician, nursing staff, or supplying pharmacist) who was primarily involved in the resolution of the DRP was counted for evaluation.

### Sample size

According to an internal previous study in a similar setting, we hypothesised that in 70% of the patients a medication change would occur during the respective study time. We considered a relative reduction of at least 29–30% due to the pharmaceutical intervention as clinically relevant (i.e. a rate of medication changes of 49–50% of patients in the intervention group). Hence, for the primary endpoint a sample size of at least 85–93 residents per group would need to be included using a two-sided chi-square test at a significance level of a = 0.05 and a power of 1 - b = 0.80.

### Data analysis

Analysis and data management were performed by using IBM SPSS Statistics Version 26.0 (IBM Corporation, Armonk, New York, USA) and Microsoft Office Excel (Microsoft Corporation, Redmond, Washington, USA). *P* values ≤0.05 were considered statistically significant. *P* values for secondary outcomes were not adjusted.

#### Outcome comparison

Statistical analysis of the residents’ characteristics was performed by using the Mann-Whitney U test for non-parametric data or the chi-square test for dichotomous data. Primary (medication changes) and secondary endpoints (medication changes in subcategories, chronic medications, hospital admissions, falls and deaths,) were tested at t1 and t2 with the chi-square test or Fisher’s exact test as appropriate.

#### Medication review procedure

The medication review procedure in the IG was evaluated descriptively and included the following steps:

- DRP identification: Identified DRPs were assigned to the predefined main and subcategories.
- Pharmacist recommendations: Relevant forwarded pharmacist recommendations were evaluated regarding the involved healthcare professionals. DRPs that were assessed as not clinically relevant after the pharmacist evaluation, were not forwarded to healthcare professionals and therefore excluded for further analysis.
- Healthcare professionals` feedback on pharmacist recommendations: The feedback was categorised in 5 categories modified based on the PCNE classification [6]: ‘Pharmacist recommendation accepted’, ‘Pharmacist recommendation not accepted’, and ‘Pharmacist recommendation noted without feedback’. DRPs that remained unclear after contact with all healthcare professionals involved were labelled as ‘DRP requires further review after feedback’. This could have been the case, for example, when unclear laboratory data had to be further evaluated. If the DRP could be directly clarified without further required action, it was assessed as ‘DRP clarified, without the need for any further measures’. The cause for this was missing information, such as whether PRN medications were administered. Additionally, reasons for not accepted recommendations were collected in free text answers and categorised post-hoc. Multiple categories of reasons were possible for each pharmacist recommendation.
- Resolved DRPs: Medication records were analysed and checked for implemented pharmacist recommendations regarding resolved DRPs at t2. We decided to use this procedure to ensure that physicians had sufficient time to actually implement the pharmacist recommendations. DRPs were categorised in 4 categories and modified in accordance with the PCNE classification [6]: ‘DRP resolved’, ‘DRP not resolved’ and ‘DRP not assessable because of death or transfer’. DRPs that could be clarified and did not lead to any further medication changes until t2 were assessed as resolved. If a recommendation could not be verified, it was evaluated as ‘DRP not verifiable’. These not verifiable DRPs were cases where monitoring was recommended, such as electrocardiogram or potassium level checks, but could not be evaluated conclusively due to a lack of data access.

## Results

### Patient characteristics

**Fig. 1:**
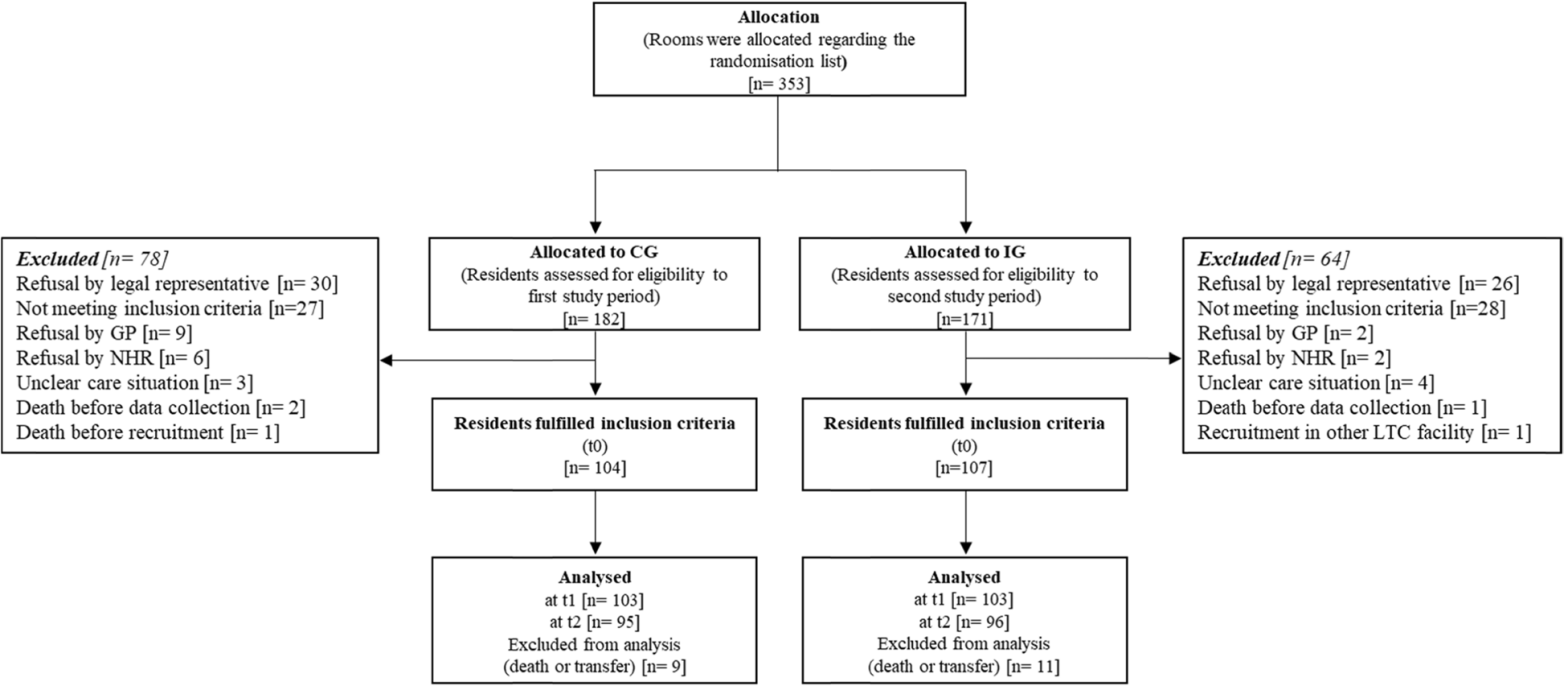
Flow chart of recruitment and lost to follow-up of NHRs during consecutive data collection. Abbreviations: *CG* control group, *GP* general physician, *IG* intervention group, *LTC* long-term care, *NHR* nursing home resident

One hundred four residents in the CG and 107 residents in the IG were included in the study (Fig. 1). We found no differences between the characteristics of residents in either study group (Table 2).

**Table 2:** Patient characteristics in CG and IG at baseline (t0)

Characteristics	CG	IG	***p*** value
Residents, total [n]	104	107	–
Residents, ownership by private association (5 wards), [n (%)]	40 (38%)	41 (38%)	0.983
Residents, municipal ownership (3 wards), [n (%)]	30 (29%)	34 (32%)	0.644
Residents, welfare ownership (3 wards), [n (%)]	34 (33%)	32 (30%)	0.663
Woman, [n (%)]	75 (72%)	72 (67%)	0.446
Age (years), median (Q25/Q75; Min-Max)	86 (78/90; 66–101)	86 (81/90; 66–100)	0.653
Length of residence (months), median (Q25/Q75; Min-Max)	31 (12/63; 1–414)	28 (12/58; 1–397)	0.437
Residents eligible alone for consent, [n (%)]	29 (28%)	34 (32%)	0.537
Residents required support for drug intake by nursing staff, [n (%)]	61 (59%)	66 (62%)	0.653
Residents with percutaneous endoscopic gastrostomy tubes (drugs must be administered by tube), [n (%)]	2 (2%)	1 (1%)	0.544
No. of chronic medications, median (Q25/Q75; Min-Max)	8 (6/10; 2–18)	7 (5/10; 2–16)	0.210
No. of PRN medications, median (Q25/Q75; Min-Max)	2 (1/3; 1–6)	2 (1/3; 1–8)	0.399
Documented diagnoses, median (Q25/Q75; Min-Max)	15 (10/21; 3–35)	14 (8/20; 3–37)	0.257
Dementia, [n (%)]	69 (66%)	69 (64%)	0.776
Diabetes, [n (%)]	41 (39%)	48 (45%)	0.424
Hypertension, [n (%)]	82 (79%)	87 (81%)	0.654
Renal failure, [n (%)]	24 (23%)	26 (24%)	0.835
Fecal incontinence, [n (%)]	16 (15%)	19 (18%)	0.643
Urinary incontinence, [n (%)]	39 (38%)	31 (29%)	0.188

*Abbreviations*: *Q25* first quartile, *Q75* third quartile, *CG* control group, *IG* intervention group, *Min* minimum, *Max* maximum

#### Outcome comparison

More medication changes (primary outcome) occurred in the IG with 78 residents at t1 compared to 52 residents in the CG (*p* = 0.001). At t2 (without continued pharmacist’s medication reviews) no differences were found (*p* = 0.680).

Table 3 shows the medication changes in total and in the corresponding subcategories. The median number of chronic medications shows a statistical difference between the two groups at the end of the second follow-up (t2: *p* = 0.005) with 9 chronic medications in the CG vs. 7 in the IG. Deaths, falls, and hospital admissions did not reach statistical significance.

**Table 3:** (i) Outcome assessment of CG and IG to both follow up measurements (t1 and t2)

	t1 compared to t0			t2 compared to t0		
Outcomes***:*** No. of primary and secondary outcomes [n]; No. of affected NHRs [n(%)]	CG; NHRs [***n*** = 103]^**a**^	IG; NHRs [***n*** = 103]^**a**^	***p*** value	CG; NHRs [***n*** = 95]^**a**^	IG; NHRs [***n*** = 96]^**a**^	***p*** value
***Primary outcome***						
No. of total medication changes	118; 52 (50%)	250; 78 (76%)	< 0.001^b^	336; 81 (85%)	385; 87 (90%)	0.680
***Secondary outcomes***						
*Medication changes in subcategories*						
No. of new drugs	46; 34 (33%)	43; 29 (28%)	0.450	120; 56 (59%)	85; 47 (49%)	0.218
No. of discontinued drugs	36; 21 (20%)	93; 49 (48%)	< 0.001^b^	122; 53 (56%)	123; 57 (59%)	0.722
No. of drugs with dose reduction	10; 9 (9%)	30; 25 (24%)	0.003^b^	24; 20 (21%)	43; 30 (31%)	0.196
No. of drugs with dose increase	10; 8 (8%)	6; 6 (6%)	0.580	15; 12 (13%)	21; 15 (16%)	0.694
No. of acute intermediate prescriptions (new and discontinued drugs)^c^	8; 7 (7%)	16; 10 (10%)	0.774	28; 22 (23%)	29; 19 (20%)	0.360
No. of documentation changes in medication records	2; 2 (2%)	36; 26 (25%)	< 0.001^b^	5; 5 (5%)	44; 28 (29%)	< 0.001^b^
No. of drugs changed to PRN medications	1; 1 (1%)	10; 10 (10%)	0.010^b^	2; 2 (2%)	13; 13 (14%)	0.005^b^
No. of altered drug regime (timing, formulation or dosage interval)	1; 1 (1%)	8; 5 (5%)	0.211	5; 5 (5%)	14; 9 (9%)	0.267
No. of other changes in the medication charts^d^	4; 3 (3%)	8; 8 (8%)	0.065	15; 13 (14%)	13; 11 (11%)	0.817
*Other secondary outcomes*	**t1**			**t2**		
No. of chronic medications, median (Q25/Q75; Min-Max)	8 (6/10; 2–19)	7 (5/10; 2–16)	0.138	9 (7/11; 2–21)	7 (5/10; 2–19)	0.005^b^
No. of falls	17; 13 (13%)	20; 20 (19%)	0.128	59; 33 (35%)	59; 39 (41%)	0.376
No. of hospital admissions	14; 13 (13%)	16; 16 (16%)	0.548	36; 29 (31%)	38; 31 (32%)	0.783
No. of deaths	1 (1%)	3 (3%)	0.621	8 (8%)	10 (10%)	0.668

*Abbreviations: Q25* first quartile, *Q75* third quartile, *CG* control group, *IG* intervention group, *Min* minimum, *Max* maximum, *NHR* nursing home resident, *PRN* pro re nata

^a^ Percentages of residents refer to the respective total number of residents at t1 or t2

^b^ *p* ≤ 0.05

^c^ Medications that were newly started and then discontinued (e.g., antibiotics needed in acute cases)

^d^ Other changes in the medication charts regarding changes that are not covered by the other categories (e.g., switch PRN medication to chronic medication)

#### Analysis of the medication review procedure

##### DRP identification

In total, 1252 DRPs with a median of 10 DRPs (first/third quartile [Q25/Q75]: 6/ 14; minimum - maximum: 2–39) per resident were identified in all 107 enrolled residents in the IG (Table 4).

**Table 4:** (ii) Overview of DRP categories of the medication review procedure in the IG

DRP categories	1) No. of identified DRPs [n]^**a**^; No. of affected NHRs [n]	2) No. of forwarded DRPs [n]^**b**^; No. of affected NHRs [n (%)]^**d**^	3) No. of resolved DRPs [n]^**c**^; No. of affected NHRs [n [%)]^**e**^
***Prescribing problems***	922; 105	721; 101 (96%)	365; 86 (85%)
Drug-drug interaction	376; 92	205; 75 (82%)	136; 61 (81%)
Unclear indication for drug	179; 81	172; 78 (96%)	99; 60 (77%)
Insufficient drug treatment	138; 77	133; 74 (96%)	30; 21 (28%)
PIM	116; 68	108; 65 (96%)	45; 33 (51%)
Dosing problems	37; 27	35; 25 (93%)	17; 14 (56%)
Inappropriate drug for indication or concomitant condition	26; 22	23; 20 (91%)	13, 11 (55%)
Therapeutic duplication	23; 18	20; 16 (89%)	11; 8 (50%)
Inappropriate treatment duration/ drug form/ administration time	19; 18	17; 17 (94%)	9; 9 (53%)
Contraindication	8; 6	8; 6 (100%)	5; 4 (67%)
***Medication use problems***	196; 68	182; 64 (94%)	108;48 (75%)
Incorrect storage	71; 46	68; 43 (93%)	51; 34 (79%)
Incorrect dispensing	61; 38	59; 36 (95%)	27; 22 (61%)
Incorrect administration	48; 33	41; 29 (88%)	21; 18 (62%)
Incorrect preperation	16; 8	14; 7 (88%)	9;4 (57%)
***Documentation problems***			
Incomplete or unclear documentation in medication records	134; 62	127; 60 (97%)	53; 37 (62%)
***Total***	**1252; 107**	**1030; 103 (96%)**	**526; 94 (91%)**

*Abbreviations: DRP* drug related problem, *NHR* nursing home resident, *PIM* potentially inappropriate medication

^a^ Data of all identified DRPs includes both clinically relevant and not clinically relevant DRPs

^b^ Data of forwarded DRPs includes only the clinically relevant DRPs that were forwarded to the healthcare professionals

^c^ Data of resolved DRPs includes only the clinically relevant DRPs and refers to the DRPs forwarded to healthcare professionals

^d^ Percentages of residents refer to the respective number of residents with identified DRPs (1)

^e^ Percentages of residents refer to the respective number of residents with DRPs forwarded to healthcare professionals (2)

##### Pharmacist recommendations

**Fig. 2:**
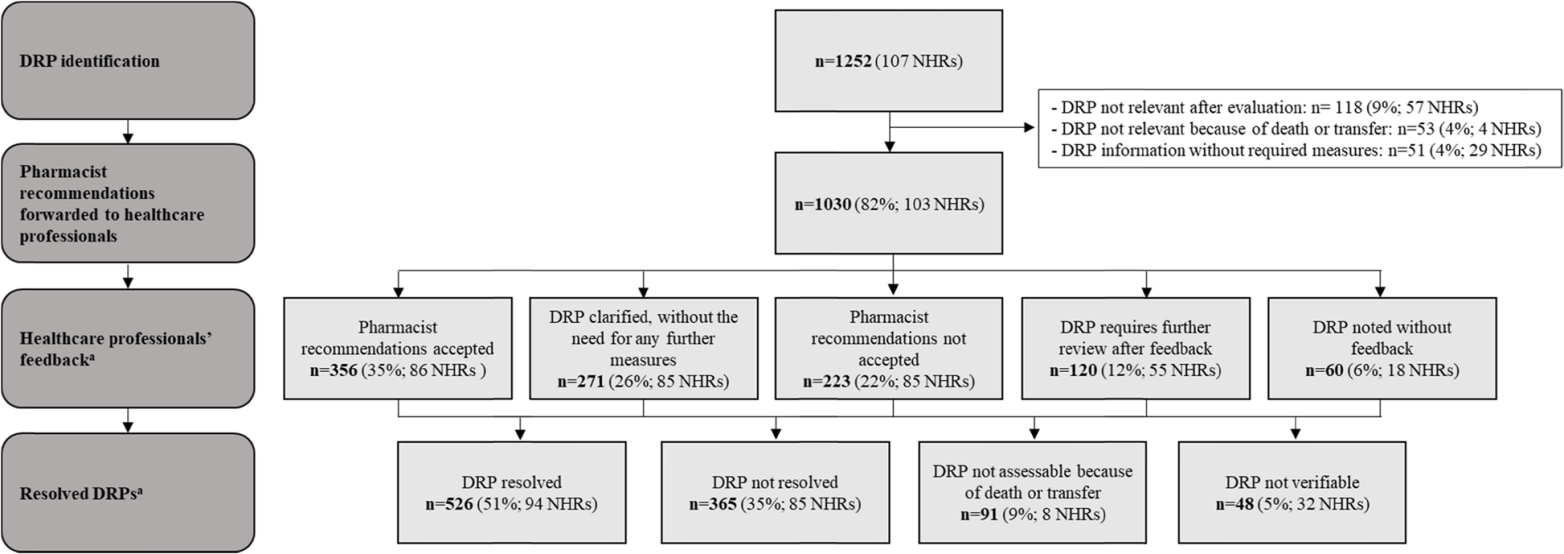
Overview of DRPs in the medication review procedure in the IG. Abbreviations: *DRP* drug related problem, *IG* intervention group, *NHR* nursing home resident. ^a^ Percentages of DRPs refer to all forwarded DRPs to healthcare professionals [*n* = 1030]

From 1252 identified DRPs, 1030 (82%) were determined to be relevant and resulted in recommendations being forwarded to the healthcare professionals (Table 4, Fig. 2). Overall, the pharmacist contacted 28 different GPs, 17 nurses, 11 different specialists (8 neurologists, 2 dermatologists and 1 ophthalmologist) and 4 community pharmacies, mostly through personal contact for 82% (846 DRP s) of all forwarded recommendations.

##### Healthcare professionals’ feedback on pharmacist recommendations

**Fig. 3:**
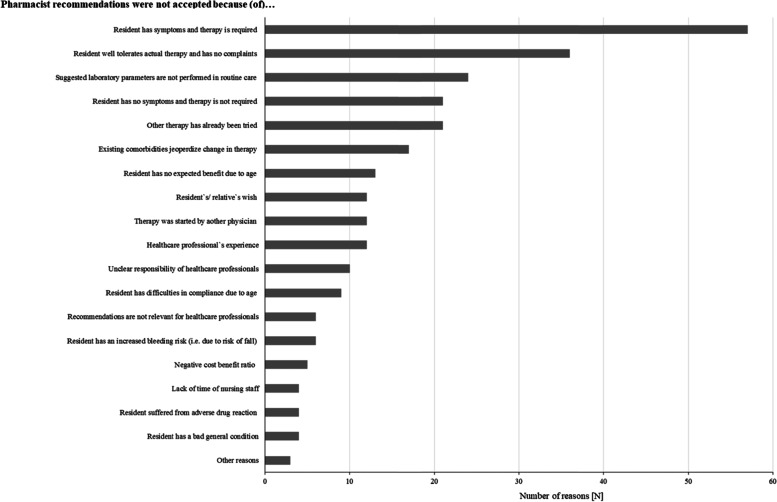
Number of not accepted pharmacist recommendations and respective reasons. Multiple categories of reasons per DRP possible [n total = 276]

Figure 2 shows the feedback of the healthcare professionals. In total, feedback was received in 94% (970 DRPs) of 1030 forwarded pharmacist recommendations, with 61% accepted (35%; 356 DRPs) or clarified DRPs (26%; 271 DRPs). 22% (223 DRPs) were not accepted. 12% (120) of DRPs required further review and 6% (60 DRPs) remained without feedback. For non-acceptance, 276 different reasons were given (Fig. 3). With 21% (57 reasons) the reason named most frequently was that residents had symptoms and required the therapy due to a risk-benefit assessment. In this case, the three most involved drugs were the antipsychotics melperone (*n* = 25), quetiapine (*n* = 10) and pipamperone (*n* = 3).

##### Resolved DRPs

Of all forwarded 1030 DRPs, 51% (526) were resolved at t2 (Table 4). Non-resolved DRPs were counted in 35% (365) of DRPs. Eight residents died or moved to another LTC facility; therefore, 9% of forwarded DRPs (91) could not be finally evaluated. A further 5% of DRPs (48) were determined as ‘not verifiable’ (Fig. 2).

## Discussion

Our multicentre, controlled intervention study of LTC residents analysed the impact of a one-time, pharmacist-led medication review on patient-relevant outcomes such as actual medication changes, number of chronic medications, hospital admissions, falls, and deaths. Compared to the CG, our intervention resulted in significantly more medication changes in the IG. Primarily, medication was discontinued. We assume that the intervention thereby contributes primarily to discontinuing unnecessary medication. However, no significant improvement was observed for hospital admissions, falls and deaths after the one-time intervention.

The medication review performed in the IG identified a high prevalence of DRPs suggesting the need to incorporate these services within LTC residents. The reconsideration of DRPs with the physicians, nurses and community pharmacists provided missing information resulting in DRP clarification and further essential reasons for not-accepted recommendations, with the requirement of therapy treatment as the most mentioned reason. Thus, it can be assumed that the intervention led to a responsible and professional reconsideration of whether the patients’ medication was essential or not. Half of all forwarded DRPs in the IG could be resolved by the procedure.

We found a higher amount of medication changes in the IG compared to the CG. This is line with another previous randomised controlled study in which a pharmacist-led medication review also led to a significantly higher medication change rate compared to a CG [19]. A differentiated look at the level of our analysed subcategories contributes to assessing the intervention’s success more precisely. Those results revealed that differences were more likely to be found in subcategories that indicated a reduction of medication (e.g., discontinued drugs), whereas an increase of drugs (e.g., new drugs) showed no differences. Based on the differentiated mentioned reasons for not accepting pharmacist recommendations, it can be presumed that physicians usually discontinued unnecessary medications due to the intervention, which supports the importance of deprescribing harmful medicine in this setting [20]. This is also supported by significantly more drugs being prescribed in our CG than in the IG at the second follow-up period. Those results further indicate that the medication review contributes to protect patients from overprescribing that causes complex drug-drug interactions and adverse events [31] as well as unnecessary prescribing cascades [32].

Interestingly, the number of medication changes was not sustainable in the second follow-up period in which no intervention was performed. This can be explained by the fact that the medication review was performed only once. Therefore, it is also reasonable to assume that this was not frequent enough to have an impact on secondary outcomes, such as hospital admissions, falls, and deaths.

The present findings seem to be consistent with those of previous reviews that reported the lack of evidence for health improvements through interventions in this setting [18, 33]. Therefore, this study, which investigated a one-time medication review, underlines the need for effective strategies such as repetitive medication reviews, for a better understanding of factors that can have an influence on patient-relevant outcomes in this frail collective and it emphasises the need for well-established, large scale, controlled studies.

The medication review procedure in the IG identified a median of 10 DRPs per resident, which lies within the range of previous studies, depending on the methodology and the DRP classification system [16, 34–36]. Our results demonstrate that the DRP categories ‘drug-drug interaction’, ‘no clear indication for drug’ and ‘insufficient drug treatment’ were most common for prescribing problems. These DRP categories seem to be especially relevant and were described by others [16, 35]. Therefore, these results should sensitise all involved healthcare professionals in LTC facilities to pay special attention to these reported DRPs in their daily routine. All these studies revealed prescription problems; however, further DRPs in medication use were not entirely covered. Our findings demonstrate that in addition to prescribing problems, residents were affected by storage problems, as well as problems with dispensing, preparing, and administering medication. Particular attention should be paid to the need for modification of medication to facilitate swallowing in dysphagia by crushing tablets or opening capsules [37–39]. The resulting risks for adverse outcomes such as an increased toxicity or decreased efficacy [40] are preventable through valuable advisory services provided by pharmacists [41]. In addition, the administration of frequently complex medication regimes and concomitant modifications of medications can often be burdensome for the residents and costly in terms of nursing time. Here, the involvement of pharmacists can make another important contribution to optimising and simplifying medication by giving recommendations, such as administering medications at the same time or standardising routes of administration. These can lead to savings in cost and nursing time and offers benefits for residents in terms of an improved quality of live [42]. Furthermore, our study highlights the relevance of incomplete documentation which was described as one of the most common error types in medication records in this setting [43].

Multidisciplinary approaches are a key for successful medication reviews [44]. Therefore, the current medication review procedure not only addressed physicians but also involved nurses and supplying pharmacies. This finally resulted in 51% resolved DRPs, which is in the range of other studies that had implementation rates of 33% up to 92% [15, 35]. The multi-professional exchange on pharmacist recommendations in the present study was realised predominantly through face-to-face meetings leading to a high feedback rate of 94%, compared to another study that used written recommendations that were less effective [34]. Regardless of whether measures were actually implemented and the DRPs were thus deemed to have been resolved, one third of the recommendations were initially accepted by the healthcare professionals. This is comparable to other studies that reported variances of 9% up to 76% acceptance [19, 34]. In one additional 26% of recommendations in our study, DRPs were clarified directly without further action needed. This was especially the case when missing information on the actual use of a PRN medication, laboratory values or current diagnoses had not been available. These data about the subdivision between accepted and clarified DRPs are usually not available. Therefore, it can be assumed that the number of our accepted recommendations is not comparable with other studies. Our aim was to present a distinction, in particular, a more detailed explanation for clarified DRPs, most of which were due to an underlying lack of information when assessing DRPs in LTC facilities. The multi-professional exchange on DRPs achieved access to this important clinical information and demonstrated the relevance of a closer collaboration. Better access to relevant health information can lead to more effective pharmacist recommendations. This access could be improved even more by digital solutions [45].

Furthermore, it seems that the pharmacist recommendations were not automatically implemented by the healthcare professionals but rather carefully weighed as to whether actual changes were appropriate or not. Therefore, explanations by the healthcare professionals for not accepting recommendations provided helpful factors that should be considered when conducting medication reviews in this complex patient collective. The findings suggest that healthcare professionals focussed on weighing the benefits against potential harms of a change in therapy. Such a risk-benefit assessment was particularly evident when antipsychotic drugs had been prescribed for patients with persistent agitation or behavioural problems; this was the most frequently mentioned reason for the required therapy. The finding underlines the problematic use and difficult withdrawal involved in this drug class [46, 47]. In general, it demonstrates the complexity of medication therapy in multimorbid LTC residents, who represent a particular challenge for all involved healthcare professionals, especially in terms of deprescribing. Multi-professional evaluation of a resident’s individual health and therapy could enable more efficient and precise drug therapy. It also can help to overcome the issue of insufficient information sharing and the lack of awareness of each other’s competences [48, 49].

### Limitations

A limitation is that the study was performed with a manageable patient collective, making it difficult to identify improved clinical outcomes. Nevertheless, significant differences were seen in the primary endpoint even if generalisability is difficult to guarantee. One researcher (ML) performed the medication review, data collection and the analysis. To minimise bias, a structured approach was followed that included checklists, the separation of analysis from intervention and discussion of DRPs with a second pharmacist. Although prescribing DRPs were based on validated classification systems, the recently used DRP list was not validated. This was because no validated DRP list exists for medication reviews that also addresses comprehensive medication use problems and documentation problems in LTC facilities. To increase quality, these additional DRP categories were developed by the expert panel. A further limitation is that the observation of drug handling was focussed on peroral medication and was limited to a one-time monitoring due to the organisational processes in the LTC facilities. Three facilities did not allow full-day monitoring, which would have been necessary to cover all possible handling problems by different nurses and further administrations such as inhalational drugs or transdermal drugs that have considerable risks [50]. Nevertheless, many DRPs in the medication use were identified and resolved in this study.

## Conclusions

Our controlled intervention study based on a pharmacist-led medication review resulted in more medication changes in the IG compared to the CG. Most changes were related to discontinuing medications. Reasons for not accepting recommendations show that in most cases, therapy was required. The results support the intervention’s success in discontinuing unnecessary medication. The study further identified a high prevalence of DRPs with half of them resolved through multi-professional exchange, which underlines the need for more routine implementation of medication reviews in this setting.

## Data Availability

The dataset used and/or analysed during the current study are available from the corresponding author on reasonable request.

## Abbreviations

CG: Control group
DRP: Drug related problems
GP: General practitioner
IG: Intervention group
LTC: Long-term care
Min: Minimum
Max: Maximum
PCNE: Pharmaceutical Care Network Europe
PIM: Potentially inappropriate medication
PRN: Pro re nata
Q25: First quartile
Q75: Third quartile
SmPC: Summary of Product Characteristic
